# Predicting tuberculosis relapse based on 28-day CFU, RS ratio, and/or drug contribution for novel regimens in the relapsing mouse model

**DOI:** 10.64898/2026.07.27.740024

**Published:** 2026-07-30

**Authors:** Rob C. van Wijk, Belen P. Solans, Linda Chaba, Sylvie Sordello, Anna M. Upton, Eric L. Nuermberger, Gregory T. Robertson, Nicholas D. Walter, Radojka M. Savic

## Abstract

Treatment shortening in tuberculosis therapy is needed, but testing all novel antibiotic combinations is unfeasible. Especially the tuberculosis relapsing mouse model is time- and resource demanding. Therefore, our objective is to develop a computational model predictive of long-term relapse prevention in mice based on short-term biomarkers, increasing the number of regimens that can be tested and prioritize regimens for further development. The innovative ribosomal RNA synthesis (RS) ratio is utilized to characterize drug effect on Mycobacterium tuberculosis health and activity, together with colony forming units (CFU) in murine lungs. Nine datasets of 58 unique regimens with 843 short-term biomarker and 2,239 long-term relapse observations were leveraged for model development in 3 iterations with external validations. The final model included therapeutic predictors, such as CFU and RS ratio change from baseline, and corrected for experimental conditions, to enable unbiased ranking of regimens between experiments. Model performance was optimal without model structure change despite fully separate model development at each iteration. Final external validation had an area under the receiver operator curve of 0.90. Challenging the model by assessing removal of either biomarker showed that performance of CFU only was similar to CFU and RS ratio once the sterilizing contribution of individual drugs to the regimens was accounted for. New drugs without this contribution quantified could benefit from RS ratio determination to predict relapse. Our predictive model can successfully differentiate between 2-, 3-, and 4-month regimens in the relapsing mouse model based on 4-week data only, supporting acceleration of treatment-shortening regimen development.

**One Sentence Summary:**

Our predictive model ranks new drug regimens by tuberculosis relapse prevention based on 28-day CFU and RS ratio, or on CFU only for known drugs.

## Full Text Availability

The license terms selected by the author(s) for this preprint version do not permit archiving in PMC. The full text is available from the preprint server.

